# Abnormal growth of the proximal femur due to apophyseal-epiphyseal coalescence resulting in coxa valga—a report of two cases in adolescents

**DOI:** 10.3109/17453674.2011.584210

**Published:** 2011-09-02

**Authors:** Peter A A Struijs, Roelof-Jan Oostra, Rick R van Rijn, Philip P Besselaar

**Affiliations:** ^1^Department of Orthopaedic Surgery; ^2^Department of Anatomy and Embryology; ^3^Department of Radiology, Emma Children's Hospital, Academic Medical Centre Amsterdam, the Netherlands

We present 2 cases (male adolescents) with a radiologically evident apophyseal-epiphyseal coalescence in the proximal femur, resulting in a coxa valga with a horizontal growth plate. We discuss the developmental mechanism and possible clinical relevance of this rare phenomenon.

## Case 1 (Figure 1)

A 13-year-old boy presented at our outpatient clinic with longstanding discomfort in both groins and thighs. Pain was provoked by activity and he had therefore abandoned playing soccer. There was no history of trauma or relevant comorbidity. Posture and gait were normal. The legs were well-aligned. Spinal motion was normal. Complaints could not be provoked by physical examination of the hips: there was pain-free and full range of motion and the femoro-acetabular impingement test was negative.

**Figure 1.**
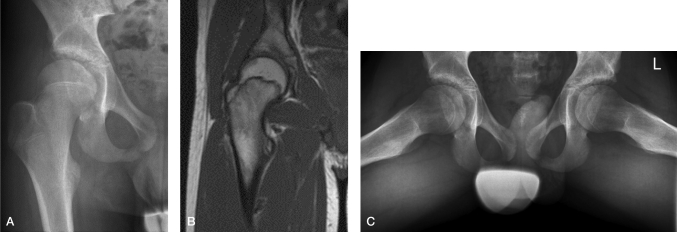
Case 1. The typical coxa valga due to coalescence (arrow) on a standard AP view (A) and T1-weighted coronal MRI of the pelvis during growth at the age of 13 years (B). On the Lauenstein view, the head-neck offset is small (C).

The radiographic AP pelvis view showed coxae valgae with well-centered spherical femoral heads. On both sides, the acetabulum was slightly shallow; the epiphysis was continuous with the apophysis of the greater trochanter, the orientation of the growth zone of the femoral neck was horizontal (transversal), and the growth zones were closing. On the Lauenstein view, the projected epiphyseal height on both sides was less than normal, as was the radial expansion of the craniolateral and anterior part of the head. Magnetic resonance imaging confirmed a (pseudo-) coalescence between the epiphysis of the femoral head and the apophysis of the greater trochanter on the right side. On the left side, this was less marked

## Case 2 (Figure 2)

A 14-year-old boy presented with occasionally mild complaints of discomfort in his right groin and thigh. Complaints were provoked by activity, although he was able to continue physical education at school.

**Figure 2.**
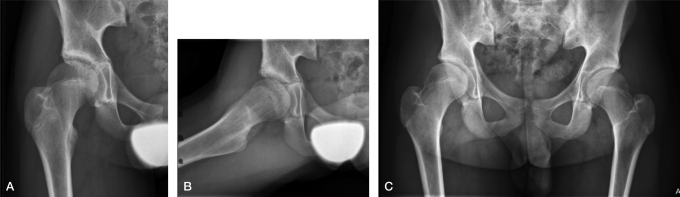
Case 2. A and B. Marked coalescence and almost absent anterior head-neck offset on standard AP (A) and Lauenstein (B) views at 14 years of age. C. AP pelvic view at 16 years of age.

**Figure 3.**
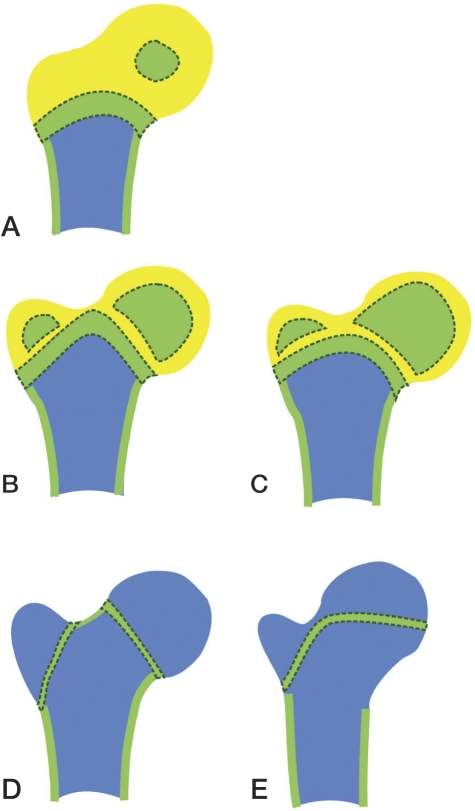
Schematic representation of the growing human hip region, showing the primary femoral and secondary capital and greater trochanteric ossification centers and proximal diaphyseal growth plate at ages of 1 year (A), 2–4 years (B, C), and > 4 years (D, E). Panels A, B, and D represent normal development, showing isthmic interruption of the chondroepiphysis prior to trochanteric osseous expansion, normal orientation of the capital growth plate, and normal head-neck offset. Panels C and E represent persistence of the isthmic part of the chondroepiphysis with subsequent coalescence of expanding secondary ossification centers, horizontal orientation of the capital growth plate, and reduced head-neck offset.

As in case 1, there was no history of trauma nor any relevant comorbidity. Physical examination of legs and spine was normal.

The AP pelvis view showed symmetrical coxae valgae with spherical heads well-centered in slightly shallow acetabula with an accessory bone at the cranio-lateral edge. On both sides, the epiphysis was continuous with the apophysis, with a horizontal orientation of the physes. On the Lauenstein view, the epiphyseal height seemed almost normal, but the craniolateral and anterior radial expansion of the head seemed to be reduced. No additional imaging was done.

In both cases, we were not able to relate the mild complaints to these abnormal radiological findings; nor could we otherwise objectify these complaints. At follow-up, complaints appeared to be self-limiting.

## Discussion

We present 2 male adolescents with a coalescence of the secondary ossification centers of the proximal femur, resulting in a coxa valga with a horizontal growth plate.

The growth pattern seems to be inborn: in contrast to the cases described by Lütken (1961), the history of our patients does not reveal any secondary cause such as past treatment for developmental dysplasia with dislocation of the hips or Perthes' disease.

In mammals, normal development of the proximal femur starts with the formation of a single chondroepiphysis. After birth, secondary ossification centers emerge both in the femoral head (epiphyseal) and greater trochanter (apophyseal). In humans, these ossification centers remain separated during further growth.

In various other mammals, these ossification centers coalesce into a single epiphysis covering the entire proximal femur, resembling the normal growth of the human proximal humerus. Lütken (1961) was the first to describe this kind of growth in the human proximal femur, pointing out its resemblance to the growth of the proximal femur in the polar bear.

Growth zones are responsible for postnatal longitudinal growth. Although commonly referred to as epiphyseal growth plates, this is a misnomer since chondrocyte proliferation and osseous transformation exclusively occur at the side facing the diaphysis (Siegling 1941). Thus, these are continuations of primary ossification centers. Secondary ossification centers have their own growth zones, which have a spherical shape, thus producing radial expansion. In all mammals, the development of the proximal end of the femur includes the formation of 2 secondary epiphyseal ossification centers in a single chondroepiphysis that is continuous with the resting cartilage of the diaphyseal growth plate. These centers, which will give rise to the femoral head and the greater trochanter, appear in man during the first year and from the third year of life, respectively (Scheuer and Black 2004). In humans and also in hominoids, marine mammals, several rodent species, and tree shrews these centres normally remain separate until the end of growth whereas in carnivores (polar bears), ungulates, proboscidae, marsupials, and several primate species the 2 centers coalesce at some time during postnatal development to continue as a single halter-shaped epiphysis. Rather than with phylogeny, body size, or locomotion type, the ossification patterns appear to correlate with the species-specific shape of the proximal femur (Serrat et al. 2007, Hogervorst et al. 2009).

Thus, in normal human growth, the central part of the chondroepiphysis in-between the capital epiphysis and the greater trochanteric apophysis does not ossify, but thins more and more during further growth. Perhaps the chondroepiphysis in this area even becomes discontinuous at some point during childhood. Anyhow, this development does not limit the underlying growth plate, which contributes to some femoral length but more particularly to the shape of the proximal end of the femur—specifically the central zone in-between the growth zones of the femoral neck and the greater trochanter (Siffert 1981).

During childhood, the growth plate of the neck gradually becomes orientated perpendicular to the compression force on the femoral head. This growth plate is responsible for the neck length (Serrat et al. 2007), thus influencing femoral offset, freedom of motion, and a lever arm for the abductor muscles. The growth plate of the greater trochanter has to resist the traction force of these abductor muscles, and also diverges during growth, but less from the longitudinal axis of the femur—thus also contributing to the lever arm. The growth zone at the isthmus contributes to the growth of the cranial part of the neck until the end of growth (Ogden et al. 1987).

In the cases we present, the in-between part of the chondroepiphysis did persist, thus enabling the expansively growing ossification centers of the greater trochanter and the femoral neck to coalesce at some time during childhood—probably in early pubertal growth. Consequently, the proximal femoral growth zones become limited by the overlying bone plate, which acts as a bracket or tether. This will not compromise longitudinal femoral growth but does alter the shape of the proximal femur: growth at the craniolateral part of the neck is inhibited whilst growth at the mediocaudal part is not.

As a result, the growth zone of the femoral neck is tethered into a horizontal (transversal) plane and the femoral head into a valgus position, leading to secondary dysplasia with a relatively small femoral offset and short lever arm. The radial expansion of the craniolateral and anterior part of the femoral head is inhibited, resulting in a smaller spherical area at this part of the femoral head and a smaller head-neck offset as seen on the AP and Lauenstein views in our cases, especially case 2. This phenomenon has also been described by Siebenrock et al. (2004) and might have clinical implications such as femoral-acetabular impingement. The formation of an apophyseal-epiphyseal coalescence shows similarities to epiphyseal brackett formation in the metatarsals. In this entity, deformity occurs through a C-shaped proximal epiphysis situated along the medial side of the diaphysis, which extends toward both the proximal and distal epiphyses. This causes abnormal growth of the bone, resulting in progressive shortening and angular displacement (Light and Ogden 1981, Ogden 1981). Tethering occurs similarly to the proximal femoral coalescence as previously described.

In contrast to the clinical cases described by Lütken (1961), the histories of our patients did not reveal any secondary cause for this kind of growth such as past treatment for developmental dysplasia of the hips, Perthes' disease, (chronic) arthritis, or (repetitive) trauma. From this, and all the more because this kind of growth is similar to the growth pattern seen in certain mammal species as mentioned before, we consider this growth not to be a developmental disturbance but a late manifestation of an inborn anomaly—or even a genetically determined abnormal growth variant. Abnormal not only because of its rarity, but particularly because it results in a secondary dysplasia with compromising sequelae: the relatively small lever arm which is mechanically less efficient, the smaller femoral offset which reduces mobility, and the smaller head-neck offset which may result in anterior femoro-acetabular impingement. These sequelae might trigger early-onset osteoarthritis. For prevention, a varus osteotomy may improve complaints, although one should realize that offset is significantly reduced in these patients.
